# Research Misconduct and Medical Journals

**DOI:** 10.1017/jme.2025.35

**Published:** 2025

**Authors:** Howard Bauchner, Robert Steinbrook, Rita F. Redberg

**Affiliations:** 1 Boston University, Boston, United States; 2 Yale School of Medicine, New Haven, Connecticut, United States; 3 University of California San Francisco, San Francisco, California, United States

**Keywords:** research, misconduct, scientific misconduct, journals and research misconduct

## Abstract

Journal editors often deal with allegations of research misconduct, defined by the Office of Research Integrity (ORI) in the United States as fabrication, falsification, and plagiarism. It is important that editors have a transparent and consistent process to deal with these allegations quickly and fairly. This process will include the authors and may include research integrity officers at the sponsoring institution as well as funders. Retractions may not be consistent with the ORI definition, for example, specifying inadequate peer-review and unreported conflict of interest, but nevertheless represent scientific misconduct.

Research misconduct threatens the validity of science, undermines trust in science, and contributes to misinformation and disinformation about science. Journals, as the conduit for research reports, review articles, and opinion pieces, play an important role in adjudicating research misconduct. Authors, editors, research integrity officers at academic institutions, and occasionally funders, are often involved in investigating questions of research misconduct.

Various definitions of research misconduct have emerged over the past decade. For the purposes of this chapter, the definition from the Office of Research Integrity (ORI), in the Department of Health & Human Services, will be used.^1^ “Research misconduct means fabrication, falsification, or plagiarism in proposing, performing, or reviewing research, or in reporting research results. (a) Fabrication is making up data or results and recording or reporting them. (b) Falsification is manipulating research materials, equipment, or processes, or changing or omitting data or results such that the research is not accurately represented in the research record. (c) Plagiarism is the appropriation of another person’s ideas, processes, results, or words without giving appropriate credit. (d) Research misconduct does not include honest error or differences of opinion.”^2^ It should be noted that image manipulation, which is often a focus of research misconduct, is a form of falsification, and although plagiarism in the form of appropriation of ideas and words is listed as a form of misconduct, in our experience (HCB, Editor in Chief of Archives of Disease in Childhood (2004-2011) and JAMA and the JAMA Network (2011-2021); RFR, Editor in Chief of JAMA Internal Medicine (2009-2023); RS, Editor at Large, JAMA Internal Medicine (2013-2023), it is unusual for plagiarism to lead to a retraction of a published manuscript. This may reflect the addition of plagiarism software in the peer review process.

It is difficult to assess how often research misconduct occurs, as it is not always detected and not tracked systematically. One approach is to determine how often published manuscripts are retracted for reasons consistent with misconduct, although this is an imperfect measure for various reasons. For example, if articles are retracted for inadequate journal peer review, it is impossible to know if research misconduct consistent with the definition of ORI occurred. The number of publications in fields including health sciences, engineering, physics, and the biological and biomedical sciences has increased substantially over the past two decades, from approximately one million per year in 1996 to three million per year in 2020, with China (23%) and the United States (16%) producing almost 40% of the publications.^3^ Thus, assessing the totality of the research enterprise is nearly impossible and does not include unpublished manuscripts, abstracts presented at research meetings, preprints, or grant applications. The 2017 National Academy of Medicine report on “Fostering Integrity In Research,” concluded that there has been a substantial increase in journal retractions due to research misconduct based on published studies from 2012-13.^4^ In a more recent study published in 2024 that assessed 24,542,394 publications indexed in PubMed between 1999 and 2022, Furuse found that six per ten thousand were retracted.^5^ The rate increased between 1999 and 2019 before declining, although it is possible that insufficient time had elapsed when the analyses were conducted to accurately assess the rate of retraction in the last few years. Retraction Watch, which is a website that includes a blog whose parent organization is the Center for Scientific Integrity, is an excellent current source for retractions, maintaining various databases, and other information regarding scientific misconduct.^6^

In some regards any estimate of the rate of retraction may be less important than high-profile examples of misconduct. For example, two of the world’s most influential medical journals, *The Lancet* and *The New England Journal of Medicine*, each retracted a research report about COVID-19 from the same company because of misconduct.^7^ Of concern is that the article retracted from *The New England Journal of Medicine* had been cited 934 times, including in preprints and non-peer reviewed papers. Of 652 verified citations, 355 occurred more than three months after the manuscript had been retracted and only 115 cited or noted the retraction.^8^ Such a large number of citations following retraction (approximately half of published manuscripts are cited less than four times) suggests that retracted research is still a source of scientific information.^9^ In July 2024, *Psychopharmacology* retracted three articles regarding MDMA therapy, also known as Ecstasy, with one accompanied by the following announcement: “The Editors have retracted this article after they were informed of protocol violations amounting to unethical conduct at the MP4 study site by researchers associated with this project. The authors have subsequently confirmed that they were aware of these violations at the time of submission of this article, but did not disclose this information to the journal or remove data generated by this site from their analysis. Additionally, the authors also did not fully declare a potential competing interest.”^10^ This statement highlights that there are numerous reasons for retraction, in this case ethical concerns and incomplete declaration of possible conflicts of interest. This retraction occurred one day after the FDA decision denying approval of MDMA for the treatment of post-traumatic stress disorder. In addition, we are unaware of any analysis of the impact of articles retracted for research misconduct on patient outcomes.

In our experience as editors, it is rare that there are professional consequences, such as loss of tenure or a professional position after findings of research misconduct resulting in one or more retractions. However, there have been some high-profile cases such as the one involving the former President of Stanford, who resigned following an independent investigation that found several published manuscripts, of which he was the senior author, did not meet scientific standards. Reportedly, he will request retraction of three manuscripts.^11^ In 2018, Brian Wansink retired from Cornell University, following the retraction of six of his articles from *JAMA* Network journals and an internal report that included: “misreporting of research data, problematic statistical techniques, failure to properly document and preserve research results, and inappropriate authorship.”^12^

## Journals and Research Misconduct

It is important for all journals to have a policy which carefully defines research misconduct and lays out a consistent stepwise approach to deal with allegations of misconduct.^13^ The same group of individuals, such as editors or integrity officers at journals or publishers, should deal with allegations of misconduct to ensure consistency and expertise. For example, at *JAMA* and the *JAMA* Network, allegations of misconduct for any of the journals were generally handled by the same group of senior editors.^14^ It is our belief that in general, while there are exceptions, journals should assume that authors did not commit misconduct, i.e., authors should be considered innocent until proven guilty.

When allegations are received, first, journals need to determine if the allegation is consistent with research misconduct or may simply be an error requiring a correction or an exchange of letters to the editor. This often requires additional information about the allegation. If the journal believes after initial review, such as analyzing the distribution of variables in a randomized clinical trial, that research misconduct may have occurred, the editors must decide if a statement — “a notice of expression of concern” — on the website indicating that the manuscript is under investigation for possible misconduct is necessary. Not all allegations require such a notification. This decision should be made on a case-by-case basis depending on the seriousness of the possible misconduct and its public health implications. For example, if there would be immediate diagnostic or therapeutic implications, a prompt “notice of expression of concern” should be posted.

Second, after these initial decisions are made, the journal should contact the corresponding author. It is important to note that the current International Committee of Medical Journal Editors (ICMJE) authorship criteria include: “Final approval of the version to be published; AND Agreement to be accountable for all aspects of the work in ensuring that questions related to the accuracy or integrity of any part of the work are appropriately investigated and resolved” to ensure that no author could indicate that they are not responsible for the misconduct and avoid assisting in any potential investigation.^15^ As authorship has changed over the years to now sometimes have multiple first, last, and corresponding authors, as well as writing teams representing dozens of authors, most journals still require that there be a single corresponding author who serves as the point of contact for the journal and is responsible for the published manuscript in its entirety.

Journals vary on whether the individual making the allegation can remain anonymous from the both the journal and the author(s). We believe that it is best if the journal is aware of who is making the allegation, since it is possible that individuals may have their own biases, which could influence how the journal handles the allegation. Most journals do not insist that the person making the allegation is identified to the authors of the manuscript in question and we do not believe that is necessary.

The allegation should be forwarded with as much detail as possible to the corresponding author. Often, it is necessary for the journal to ask for more details with respect to the allegation. If the allegation is not clear, or lacks detail, then it may not be possible for the author(s) to respond. When contacting the corresponding author, the journal should specify a deadline for the response. At this time, journals must also decide if they want to contact the author’s institution. For various reasons, including slowing the process of retraction, most journals try to resolve allegations of misconduct without contacting institutions, although several research integrity officers at institutions that we talked with prefer that they be contacted as soon as a journal decides that research misconduct may have occurred.

Concerns have been raised that it often takes too long for journals to retract manuscripts, sometimes years, and this may be in part because journals do not routinely provide deadlines.^16^ It took 12 years to retract one of the most notorious papers about the relationship between the measles, mumps, and rubella vaccine and bowel disease and autism.^17^ In our experience, many authors often ask for an extension to respond to allegations. Whether such an extension is granted should be decided on a case-by-case basis, but journals need to carefully consider how much time should be granted.

Some authors do not respond to queries about misconduct from a journal. This poses a challenge for journals. Few journals have the resources or access to the data to conduct their own detailed investigation. After the deadline has passed, an additional query to the author(s) is warranted. At this time, it may be appropriate to correspond with all the authors of the manuscript, particularly the first and last author, or to contact a research integrity officer at the sponsoring institution if that has not already been done. This approach works only if the institution has such an individual and that person can be identified. This is not always possible. Recently, journal editors and research integrity officers from three US institutions conducted a series of twelve virtual meetings with the shared goal of improving the reliability of scientific data by reducing and addressing research misconduct. This working group made three recommendations to promote collaboration between institutions and journals: “(1) reconsideration and broadening of the interpretation by institutions of the need-to know criteria in federal regulations (i.e., confidential or sensitive information and data are not disclosed unless there is a need for an individual to know the facts to perform specific jobs or functions), (2) uncoupling the evaluation of the accuracy and validity of research data from the determination of culpability and intent of the individuals involved, and (3) initiating a widespread change for the policies of journals and publishers regarding the timing and appropriateness for contacting institutions, either before or concurrently under certain conditions, when contacting the authors.”^18^

Another avenue is for the journal to contact the research funder, although we have rarely found funders to be particularly helpful in this role. Delays in retraction occur because authors can be unresponsive, disagree with the allegations, or request repeated extensions. Delays also occur because institutional integrity officers cannot be identified, or if they are identified, they also request extensions. Investigations can involve multiple authors, are sometimes quite complex, and can take months to conduct.

If an author does respond with sufficient information, then the journal must once again decide if research misconduct has occurred, and retraction is warranted. As editors we have never believed that retraction in any way diminishes a journal. Honesty is the bedrock on which research, the peer review process, and publication is based. Journals must assume that what they publish reflects an accurate representation of the research. However, when they learn that is not the case, retraction ensures that the scientific record is accurate, a paramount obligation of journals.

If the authors are not cooperative or challenge the allegation, the editors either can continue discussions with the author(s) or elevate it to a formal query to a research integrity officer and/or department chairperson of the corresponding author or the senior author. It is important that deadlines be developed for each step, otherwise investigations will be delayed unnecessarily.

If a journal decides that a retraction is necessary, it is important to post it as soon as possible. The Committee on Publication Ethics (COPE) is a non-profit organization started in 1997 with over 13,000 members whose mission is to educate and support editors and publishers to create an exemplary culture of publishing.^19^ COPE has provided a list of items that should be included in retractions:

- Be linked to the retracted article wherever possible (ie, in all online versions)
- Clearly identify the retracted article (eg, by including the title and authors in the retraction heading or citing the retracted article)
- Be clearly identified as a retraction (ie, distinct from other types of correction or comment)
- Be published promptly to minimise harmful effects
- Be freely available to all readers (ie, not behind access barriers or available only to subscribers)
- State who is retracting the article
- State the reason(s) for retraction
- Be objective, factual, and avoid inflammatory language.^20^

In 2024, the National Information Standards Organization, a United States nonprofit organization “that identifies, develops, maintains, and publishes technical standards to manage information” released specific recommendations regarding how journals should publish retractions, including how they should be titled and best practices for the metadata.^21^ We would add that if the article has been posted on a preprint server, the journal or the authors should contact the preprint server to ensure that it is noted on the preprint that the linked article has been retracted.

It is our experience that authors are increasingly acknowledging mistakes in their manuscripts and even requesting retraction. This likely reflects the complexity of contemporary research and greater awareness among investigators that it is critical to ensure that the scientific record is accurate. Journals should review these requests, determine that the authors have clearly stated the mistake and why they are requesting retraction, and in most cases, retract the manuscript. In addition, it is important that journals ensure that all authors agree with the request to retract a manuscript. If not, further investigation involving the authors’ institutions is necessary.

## Retraction and Replacement

Several journals have pioneered the use of retraction and replacement of articles.^22^ This approach has been developed to encourage authors to contact journals if, after publication, they find substantial errors in the manuscript, such as coding errors in the statistical analysis, that may require retraction. If contacted by authors, journals then have several decisions to make. First, is a correction sufficient or is retraction with replacement necessary? In general, at *JAMA* and the *JAMA* Network this decision was based on the direction or magnitude of changes in the main results, interpretations, and conclusions. If the science was still considered valid, a letter or explanation with retraction and replacement of the published article could be considered. At *JAMA*, the newly published manuscript is linked to a letter of explanation and noted as retracted and replaced. If the underlying science is no longer considered valid, then a retraction is necessary. Second, the journal must also decide, given the new conclusion, if it would have published the manuscript. If the answer is no, then retraction, not retraction with replacement, is necessary. The latter decision is subjective and often requires re-review by peer reviewers to help decide the value of the “new” manuscript.

## Other Definitions of Research Misconduct

There are other and broader definitions of research misconduct than the one from ORI. For example, COPE includes both the ORI definition as well as: “Scientific misconduct is a continuum ranging from honest errors to outright fraud. The research community must take a collective responsibility even for its deviants. Moving the whole research community in the right direction should reduce the number of serious cases.”^23^ ICMJE, which is a small working group of editors, including *BMJ*, *JAMA*, *The Lancet*, and *The New England Journal of Medicine*, states: “Scientific misconduct in research and non-research publications includes but is not necessarily limited to data fabrication; data falsification including deceptive manipulation of images; purposeful failure to disclose relationships and activities; and plagiarism. Some people consider failure to publish the results of clinical trials and other human studies a form of scientific misconduct.”^24^ The National Academy of Medicine (NAM), in their 2017 report on “Fostering Integrity In Research,” reaffirmed that fabrication, falsification, and plagiarism were central to the definition of misconduct, consistent with their earlier 1992 NAM report, but acknowledged that other definitions were more expansive. For example, the National Science Foundation (NSF) also included “other serious deviations from accepted practices in proposing, carrying out, or reporting research results from activities funded by NSF.”^25^ Importantly, the US Public Health Service recently finalized its rule, which took effect on January 1, 2025, on regulations governing research misconduct, and stated, “Research misconduct means fabrication, falsification, or plagiarism in proposing, performing, or reviewing research, or in reporting research results. Research misconduct does not include honest error or differences of opinion.”^26^

These more expansive definitions, which could be part of the definition of scientific misconduct rather than the specific definition of research misconduct used by the US Public Health Service, raise the issue of whether other deviations should be considered misconduct, such as undeclared conflicts of interest. Undeclared conflicts of interest are a challenging issue for many reasons. For example, not all journals agree regarding the reporting period of conflicts of interest, or an author may indicate not recall a conflict or have a difference of opinion about what represents a conflict of interest. It is difficult for editors to know if the omissions were intentional or unintentional. Furthermore, since journal editors usually do not share this type of information with other journal editors, and there is no national/international registry of individuals with undeclared conflicts of interest, repeat offenders are not easily identified. Journal editors could consider sharing this information, but a mechanism for sharing would need to be established. The decision on retraction of an article versus a correction for undeclared conflict of interest may depend upon the seriousness of the conflict of interest or if an author has failed to report conflicts of interest on numerous occasions. Other violations that may be considered research misconduct, although beyond the definition of ORI, include major protocol violations in studies, particularly randomized clinical trials; ethical lapses, for example not obtaining appropriate consent in prospective cohort studies; inadequate journal peer review; lack of trial registration; changing of endpoints after data unblinding; or refusal to share data when necessary. The latter is particularly complicated because sharing the data that underpins the published research report has been mandated by the White House Office of Science and Technology Policy, requiring each US federal agency that funds biomedical research to develop a detailed plan to ensure data sharing.^27^ However, most industry funded studies do not share the data on which the published research article was based. The 2017 NAM Report recommended that “medical journal and book publishers should ensure that the information sufficient for a person knowledgeable about the field and its techniques to reproduce reported results is made available at the time of publication or as soon as possible after that.”^28^ However, data sharing is currently occurring on a limited basis, in part because how sharing will be accomplished, who will fund it, and who will enforce any plan developed by US federal agencies remains unclear. NAM did recommend the establishment of an independent nonprofit organization — a Research Integrity Advisory Board (RIAB) — but seven years later, there still has been no action to accomplish this important goal. An additional concern regarding possible misconduct has arisen in opinion pieces or editorials, when authors exaggerate the quality of the evidence, or only cite some evidence, as the basis for their opinions.^29^ Whether such opinion pieces represent scientific misconduct, rather than research misconduct as defined by ORI, and should be retracted remains unclear.

We believe a uniform definition of research misconduct across institutions and journals would be helpful. We acknowledge how challenging developing such a definition would be, given the various opinions about some concepts of misconduct, for example, undeclared conflicts of interest. Regardless, journals should apply any definition of misconduct consistently, which is easier to do if the definition of misconduct is specific and detailed. For example, reaching agreement about whether the refusal to share data represent misconduct may be difficult, since some countries restrict releasing any individual patient data, anonymized or not, and biomedical companies will want to protect intellectual property and be less willing to share data.

We agree and support the 2017 NAM recommendation that all stakeholders in the research enterprise should improve and strengthen their practices and policies to respond to threats to research integrity. NAM also identified that in industry performed or industry-sponsored research, “pressures associated with regulatory approvals or commercial release may create disincentives for full data transparency or biases that favor conclusions of safety and efficacy.”^30^ The Food and Drug Administration (FDA) plays an important role as well, when reviewing such industry sponsored data for new drug and device applications. It should assure full data transparency and freedom from bias in results and conclusions, prior to approvals of new drugs and devices. In addition, the FDA has access to and reviews all individual patient data underpinning drug and device approvals and could make those data available, while protecting proprietary information, on a consistent basis. Journal editors also play an important in determining how much sponsor influence they will allow before the concern for bias is considered too great for publication. For example, in a study of a cardiac device made and funded by Abbott, “The sponsor participated in site selection and management and in data analysis.”^31^ The authors (many of whom received payments from Abbot) declined to share the data from the study on the grounds that, “The sponsor considers the data proprietary.” Two of us (RS and RFR) believe such substantial sponsor involvement in all aspects of a trial violates the principle above articulated in the NAM report of avoiding “biases that favor conclusions of safety and efficacy.” A publication free of bias is the responsibility of the investigators, journal editors, and the sponsor, regardless of the sponsor. One of the most important roles for journal editors is to maintain scientific integrity in the published literature.

## AI and Research Misconduct

Science, medicine, and society in general has entered a new era with the onset of artificial intelligence (AI). Its uses in science and medicine are evolving at an unprecedented pace. What role AI will play in detecting scientific misconduct is unclear, but some early examples suggest substantial promise. Software is now available that can detect image manipulation which contributes to a substantial number of retractions each year and is a type of research misconduct.^32^ Editors should be aware that such software is not 100% accurate. About a decade ago, concerns were raised that the distribution of baseline variables in some randomized clinical trials was mathematically impossible, suggesting research misconduct.^33^ Some journals have been using software to identify such distributions. Many journals already use software to detect plagiarism. Ultimately, AI will be used to help draft manuscripts and assist in peer review by determining if authors have adhered to the many reporting guidelines such as PRISMA, or Prospero^34^. Inevitably it will also help to detect various types of scientific misconduct. However, AI will also undoubtedly contribute to scientific misconduct, for example, when large language models are an unnamed author for submitted manuscripts. Recently, the ICMJE clarified that AI could assist in the preparation of a manuscript, but how it was used needs to be detailed in the manuscript. They also stated that AI does not quality as an author.

## Conclusion

The National Academy of Medicine in its 2017 report entitled Fostering Integrity in Research lists six core values of science — objectivity, honesty, openness, accountability, fairness, and stewardship — that are necessary to ensure that the public has faith in the research enterprise.^35^ Authors, editors, publishers, funders, research institutions, and others must commit to these principles to ensure a successful research enterprise free of distortion and bias. Allegations of research misconduct require expertise and time to adjudicate, and it is important that publishers ensure that editors have sufficient resources to conduct such investigations. Broad consensus on definitions of research misconduct and the consequences of such conduct would be helpful. Specifically journals, many of which have become major communication networks, with reach around the world through social media, must commit to processes that quickly evaluate allegations of misconduct, and correct and retract, if necessary, what they publish.
